# Treatment with anti‐neonatal Fc receptor (FcRn) antibody ameliorates experimental epidermolysis bullosa acquisita in mice

**DOI:** 10.1111/bph.14986

**Published:** 2020-03-06

**Authors:** Anika Kasprick, Maxi Hofrichter, Bryan Smith, Penelope Ward, Katja Bieber, Anthony Shock, Ralf J. Ludwig, Enno Schmidt

**Affiliations:** ^1^ Lübeck Institute of Experimental Dermatology, and Center for Research on Inflammation of the Skin University of Lübeck Lübeck Germany; ^2^ UCB Pharma Slough UK; ^3^ Swallowfield UK

## Abstract

**Background and Purpose:**

Pemphigus and pemphigoid diseases are characterized and caused predominantly by IgG autoantibodies targeting structural proteins of the skin. Their current treatment relies on general and prolonged immunosuppression that causes severe adverse events, including death. Hence, novel safe and more effective treatments are urgently needed. Due to its' physiological functions, the neonatal Fc receptor (FcRn) has emerged as a potential therapeutic target for pemphigus and pemphigoid, primarily because IgG is protected from proteolysis after uptake into endothelial cells. Thus, blockade of FcRn would reduce circulating autoantibody concentrations. However, long‐term effects of pharmacological FcRn inhibition in therapeutic settings of autoimmune diseases are unknown.

**Experimental Approach:**

Therapeutic effects of FcRn blockade were investigated in a murine model of the prototypical autoantibody‐mediated pemphigoid disease, epidermolysis bullosa acquisita (EBA). B6.SJL‐H2s C3c/1CyJ mice with clinically active disease were randomized to receive either an anti‐FcRn monoclonal antibody (4470) or an isotype control over 4 weeks.

**Key Results:**

While clinical disease continued to worsen in isotype control‐treated mice, overall disease severity continuously decreased in mice injected with 4470, leading to almost complete remission in over 25% of treated mice. These clinical findings were paralleled by a reduction of autoantibody concentrations. Reduction of autoantibody concentrations, rather than modulating neutrophil activation, was responsible for the observed therapeutic effects.

**Conclusion and Implications:**

The clinical efficacy of anti‐FcRn treatment in this prototypical autoantibody‐mediated disease encourages further development of anti‐FcRn antibodies for clinical use in pemphigoid diseases and potentially in other autoantibody mediated diseases.

**Abbreviations**
AIBD: autoimmune bullous dermatoses
COL7: type VII collagen
EBA: epidermolysis bullosa acquisita
FcRn: neonatal Fc receptor
PD: pemphigoid diseases

**What is already known**

- The neonatal Fc receptor (FcRn) controls the half‐life of IgG (auto)antibodies.
- FcRn‐deficient mice are partly protected from induction of certain autoimmune diseases.

**What this study adds**

- Anti‐FcRn treatment improves autoantibody‐mediated experimental autoimmune disease in mice.
- Anti‐inflammatory effects of FcRn inhibition are paralleled by reduced autoantibody titres.

**What is the clinical significance**

- Inhibition of FcRn has potential as a therapeutic pathway in autoantibody‐mediated diseases.

## 1 INTRODUCTION

Autoimmune bullous dermatoses (AIBD) comprise a group of diseases characterized and caused by autoantibodies against structural proteins of the skin. AIBD can be classified into pemphigus diseases, where autoimmunity towards desmosomal antigens is the underlying cause, and pemphigoid diseases with autoimmunity against antigens located along the dermal–epidermal junction (Hammers & Stanley, 2016; Kasperkiewicz et al., 2017; Liu, Li, & Xia, 2017; Ludwig et al., 2017; Schmidt & Zillikens, 2013). Despite major advances in diagnostics and treatment, they still pose a considerable therapeutic challenge. In pemphigus, the combination of the anti‐https://www.guidetopharmacology.org/GRAC/ObjectDisplayForward?objectId=2628 antibody https://www.guidetopharmacology.org/GRAC/LigandDisplayForward?ligandId=6780 with systemic corticosteroid leads to remission, off therapy, in almost 90% of the patients after 24 months, but 40% of patients experience grade 3–4 severe adverse events. Furthermore, the time to achieve complete remission is rather long, more specifically ≥6 months after initiation of treatment (Joly et al., 2017). Faster acting and safer treatment regimens are highly desirable, as are new treatments which could replace the corticosteroid component of the regimen.

In bullous pemphigoid (BP), the most common pemphigoid disease (Hübner, Recke, Zillikens, Linder, & Schmidt, 2016), patients rapidly respond to corticosteroid treatment (Joly et al., 2002). However, relapse rapidly and frequently follows corticosteroid withdrawal leading to a need for prolonged corticosteroid use in many patients, with accompanying adverse effects (Cai et al., 2014; Joly et al., 2009; Kirtschig et al., 2010). Other pemphigoid diseases, such as mucous membrane pemphigoid (MMP) or epidermolysis bullosa acquisita (EBA), are notoriously difficult to treat (Amber, Murrell, Schmidt, Joly, & Borradori, 2018; Kim, Kim, & Kim, 2011; Murrell et al., 2015), and new treatments are needed to achieve disease control.

The https://www.guidetoimmunopharmacology.org/GRAC/ObjectDisplayForward?objectId=2985 serves several functions: First, it transfers IgG from the mother to the fetus across the placenta and from the intestine into the circulation of neonates. Second, throughout life, FcRn protects IgG (and albumin) from proteolysis after uptake into endothelial cells and hence is crucial for IgG homeostasis. FcRn is also expressed by antigen‐presenting cells (APC), such as monocytes, macrophages, and dendritic cells, as well as on neutrophils. Here, FcRn functions to recycle IgG after its uptake. Furthermore, and independent of IgG recycling, FcRn expressed on APCs is important for phagocytosis of bacteria, and a role in APC processing of immune complexes has been described (Baker, Rath, Pyzik, & Blumberg, 2014). Lastly, FcRn has been implicated in the transport of autoantibodies into keratinocytes, thus contributing to the pathogenesis of pemphigus (Chen, Chernyavsky, Webber, Grando, & Wang, 2015; Roopenian & Akilesh, 2007; Stapleton, Einarsdóttir, Stemerding, & Vidarsson, 2015; Vidarsson et al., 2006).

Hence, among others, FcRn has emerged as a potential therapeutic target for AIBD (Ludwig, Kalies, Köhl, Zillikens, & Schmidt, 2013): In antibody transfer models of pemphigus, BP and EBA, lack of FcRn or β2 microglobulin expression conferred protection against disease development. This protection could, however, be overcome by administering excess amounts of pathogenic antibodies (Li et al., 2005; Liu et al., 1997; Sesarman, Sitaru, Olaru, Zillikens, & Sitaru, 2008). High doses of intravenous IgG (IVIG), a second line treatment for AIBD (Ishii, Hashimoto, Zillikens, & Ludwig, 2010), mediates its effects partially through the inhibition of FcRn in preclinical AIBD models and in patients (Amagai et al., 2009; Hirose et al., 2015; Li et al., 2005; Sasaoka et al., 2018). Based on these observations, clinical trials are currently testing the safety and efficacy of a number of FcRn‐targeting compounds (Kiessling et al., 2017) in patients with pemphigus (NCT03334058 and NCT03075904), as well as other autoantibody‐mediated diseases (NCT03075878, NCT03052751, and NCT02718716).

Here, we selected EBA as a model disease for AIBD because a murine model of EBA, based on immunization, induces a long‐lasting disease phenotype and allows for therapeutic intervention in animals with clinically manifest disease (Bieber, Koga, & Nishie, 2017; Iwata et al., 2013; Koga et al., 2018). This model system duplicates the cascade of inflammatory EBA pathogenesis, including loss of tolerance, maintenance of IgG half‐life, and autoantibody‐induced tissue pathology. EBA is caused by autoantibodies directed against type VII collagen (COL7). Clinically, EBA manifests as either mechano‐bullous disease, characterized by skin fragility, scarring, and miliae, or, in the majority of cases, as an inflammatory disease, resembling other pemphigoid diseases. In both forms, EBA is often complicated by involvement of the mucosa of the oesophagus, larynx, or eyes (Amber et al., 2018; Iwata et al., 2018; Koga et al., 2019).

Based on these considerations, we evaluated the effect of anti‐FcRn treatment in an experimental murine EBA model and subsequently evaluated the pathways associated with therapeutic efficacy.

## 2 METHODS

### 2.1 Experiments with human samples

All experiments with human samples were approved by the ethical committee of the Medical Faculty of the University of Lübeck and were performed in accordance with the Declaration of Helsinki. Skin and blood samples from patients and healthy volunteers were obtained after written informed consent was obtained. Immunoadsorption material was obtained from two patients with bullous pemphigoid (BP). BP was diagnosed based on clinical presentation, IgG, and/or C3 deposition along the dermal–epidermal junction in direct immunofluorescent microscopy from a peri‐lesional skin biopsy, detection of anti‐BP180‐NC16A antibodies by ELISA, and binding of patient IgG to the blister roof in indirect immunofluorescent microscopy using human salt split skin as a substrate (Witte, Zillikens, & Schmidt, 2018).

### 2.2 Animals

B6.SJL‐H2^s^ C3^c^/1CyJ (B6.s, https://scicrunch.org/resources/Any/search?q=IMSR_JAX%253A000966&l=IMSR_JAX%253A000966) mice develop experimental EBA after immunization against COL7 (Iwata et al., 2013). Mice obtained from a colony held at the animal facility of the University of Lübeck were housed under specific pathogen‐free conditions and provided standard mouse chow and acidified drinking water ad libitum. For all experiments, mice of both genders were used in equal distribution. Animal experiments were approved by local authorities of the Animal Care and Use Committee (Kiel, Germany) and performed by certified personnel. Animal studies are reported in compliance with the ARRIVE guidelines (Kilkenny et al., 2010) and with the recommendations made by the *British Journal of Pharmacology.*

### 2.3 Induction of experimental EBA and treatment protocol

Adult (8–12 weeks) B6.s mice at an equal sex distribution were immunized with recombinant murine COL7^vWFA2^ emulsified in http://www.titermax.com/Vaccine-Adjuvants-Norcross-GA.html (TiterMax, Norcross, USA) as previously described (Bieber, Koga, & Nishie, 2017; Iwata et al., 2013; Kasprick, Bieber, & Ludwig, 2019). Four to 10 weeks after immunization, mice were clinically evaluated weekly. Mice developing EBA skin lesions in 2% or more of the body surface area were allocated to either isotype control or anti‐FcRn murine antibody (4470) on an alternating basis. In total, 72% (*n* = 22) of immunized mice achieved this criterion within 7 weeks of immunization. After allocation, isotype or 4470 was administered at a dose of 30 mg·kg^−1^ bodyweight i.p. twice weekly. Mice were clinically examined once per week to determine the body surface area affected by EBA skin lesions (primary endpoint of the study) by an investigator not aware of the treatment given to individual mice. Blood was obtained by venepuncture at Weeks 0, 2, and 4 of the treatment period. At the same time points at and after randomization, adverse events were documented. For this, body weight, general condition, spontaneous behaviour, and (if abnormalities were observed) respiration rate and temperature were recorded and scored as outlined in Table S1. Mice were held in individually ventilated cages at the specific pathogen‐free animal facility at the University of Lübeck. During the entire lifespan, mice had free access to standard mouse chow and acidified drinking water. Regular health monitoring (every 3 months) is implemented at the facility, and during the experiment, no infections were noted. At the end of the treatment period, anaesthetized (ketamine/xylazin) mice were killed by cervical dislocation and bleeding out.

### 2.4 Histology

H&E histology, staining for IgG (polyclonal rabbit anti‐mouse IgG‐FITC, Dako, Code No. F0232), and C3 (polyclonal goat anti‐mouse C3‐FITC, abcam, number: ab182878) from mouse skin were performed as described in detail elsewhere (Kasprick et al., 2019). The immuno‐related procedures used comply with the recommendations made by the *British Journal of Pharmacology* (Alexander et al., 2018). Semi‐quantitative scoring of H&E stained sections to determine the magnitude of the dermal infiltration was performed as described (Ludwig et al., 2005). Score values 0–3 correspond to no, mild, moderate, or severe infiltration, respectively. IgG and C3 deposits in the skin were quantified by determining the signal intensity (relative grey value) at the dermal–epidermal junction using ImageJ (https://scicrunch.org/resources/Any/search?q=SCR_003070&l=SCR_003070).

### 2.5 Determination of total and mCOL7^vWFA2^‐specific IgG concentrations in serum

Serum levels of circulating total mouse IgG were determined by ELISA using mouse IgG quantification sets (Bethyl, Montgomery, Texas, USA) as described by the manufacturer's protocol using 1:50,000 diluted samples. For detection of circulating anti‐mCOL7^vWFA2^‐IgG by ELISA, each well was coated with 250‐ng recombinant mCOL7^vWFA^ in coating buffer (0.05‐M carbonate–bicarbonate buffer, pH 9.6). After blocking, diluted samples (1:20,000) were added and incubated for 60 min. Standard reference curves were established by using the mouse reference sera (pooled serum of mice 12 weeks after immunization with mCOL7C^vWFA2^; Hammers et al., 2011). For both ELISA protocols, bound antibodies were detected by HRP‐conjugated goat anti‐mouse IgG (Bethyl) and tetramethylbenzidine (Thermo Fisher Scientific). The enzymic colour reaction was stopped by 2‐M sulfuric acid (Carl Roth, Karlsruhe, Germany), and the change in OD was measured with a plate reader (GloMax™ Discover microplate reader, Promega, Mannheim, Germany) at 450 nm.

### 2.6 Determination of antinuclear antibodies (ANA)

ANA were measured using Biochips coated with HEp‐2 cells (https://www.euroimmun.de/produkte/indikationen/autoantikrper-diagnostik/rheumatologie/sle/aak-gegen-dsdns/iift/weitere-produkte.html, Euroimmun, Lübeck, Germany) as substrate and a FITC‐labelled, polyclonal donkey anti‐mouse F (ab)_2_ antibody (Jackson Immuno Research, Ely, UK). Sera were incubated on the Biochips at a 1:100 dilution. Sera from NZM2410/J mice (https://scicrunch.org/resources/Any/search?q=IMSR_JAX%3A002676&l=IMSR_JAX%3A002676) with or without proteinuria (*n* = 3 per group) from a previous study were included as positive or negative controls (Vorobyev et al., 2019). ANA were tested in all serum samples obtained at the end of the treatment period (*n* = 11 per group).

### 2.7 Determination of CD62L expression in neutrophils in mice with experimental EBA

For flow cytometry (FACS) analysis, single cell suspensions of inguinal lymph node, splenic cells, or erythrocyte free blood (containing EDTA) from mice after immunization‐induced EBA were blocked with anti‐FcRII/III blocking antibody (Miltenyi). Next, cells were incubated in 3% BSA/PBS containing various combinations of the following antibodies: FITC‐, phycoerythrin (PE)‐, or allophycocyanin (APC)‐, VioGreen‐conjugated anti‐mouse CD45 (clone 30F11), CD62L (clone MEL‐14), Gr‐1 (clone RB6‐8C5), CD11b (clone REA592), Ly6G (clone REA526), or appropriate isotype control antibodies (obtained from Miltenyi or BD). Dead cells were excluded by staining with PI (Miltenyi). Cells were first gated for singlets (FSC‐H compared with FSC‐A) and leukocytes (SSC‐A compared with FSC‐A). The leukocyte gates were further analysed for their uptake of PI to differentiate live from dead cells and their expression of CD45, taking only the live, healthy leukocyte population. Activation status of neutrophils was assessed using gating for scatter, single cells (FCS‐A vs. FSC‐W) and CD45^pos/dim^ cells (Bieber et al., 2016; Bieber et al., 2017). The mean fluorescent intensity (MFI) of GR‐1^pos^/Ly6G^pos^/CD62L^neg^ was detected using a Miltenyi MacsQuant10 and analysed with the MACSQuantify™ Software (Version 2.11 1746 19438).

### 2.8 In vitro neutrophil activation

To evaluate the effects of FcRn inhibition on neutrophil activation, a luminol (5‐amino‐2,3‐dihydro‐1,4‐phthalazindione)‐amplified chemiluminescence assay was used to measure the total of intracellular and extracellular ROS as described previously (Behnen et al., 2014). Polymorphonuclear (PMN) leukocyte (granulocyte) fraction from healthy blood donors was isolated by Polymorphprep (Axis‐Shield, Dundee, UK) sedimentation mixed with medium (RPMI, LONZA, Cologne, Germany) and was incubated with different concentrations of the anti‐FcRn antibody (100, 10, 1, and 0.1 μg·ml^−1^) or with 100 μg·ml^−1^ isotype control antibody. Neutrophils were stimulated with immobilized immune complexes consisting of BP patient immunoadsorption material and recombinant NC16A (Sitaru et al., 2007) and analysed immediately using the GloMax™ Discover microplate luminescence reader (Promega).

### 2.9 Cryosection assay

Blister‐inducing capacity of patients' autoantibodies was evaluated using the cryosection assay, an ex vivo model of autoantibody‐induced dermal–epidermal separation originally described by Gammon et al. (Gammon et al., 1982) and modified as described (Recke et al., 2010; Sitaru et al., 2002). Human skin cryosections were incubated with IgG fractions from BP immunoadsorption material for 1 hr at 37°C (dilution 1:6 in PBS) and washed once with PBS afterwards. Leukocyte suspension from healthy blood donors was isolated by dextran 500 (ROTH, Karlsruhe, Germany) sedimentation mixed with medium (RPMI, LONZA, Cologne, Germany) and was incubated with different concentrations of the anti‐FcRn antibody (100, 10, 1, and 0.1 μg·ml^−1^) or with 100 μg·ml^−1^ isotype control (both UCB Biopharma SPRL, Brussels, Belgium) for 30 minutes on ice. Subsequently, the different leukocyte fractions were incubated with skin sections for 3 hr at 37°C. After washing with PBS, slides were fixed in formalin and stained with haematoxylin and eosin. Skin sections were photographed, and split formation was determined by measuring section length and split length using ImageJ.

### 2.10 Data and statistical analysis

The data and statistical analysis comply with the recommendations of the *British Journal of Pharmacology* on experimental design and analysis in pharmacology. If not otherwise indicated, data are presented as mean ± *SEM.* For statistical analysis, Prism (Version 8, GraphPad Software, San Diego, USA, https://scicrunch.org/resources/Any/search?q=SCR_002798&l=SCR_002798) and SigmaPlot (Version 13, Systat Software GmbH, Erkrath, Germany, https://scicrunch.org/resources/Any/search?q=SCR_003210&l=SCR_003210) were used. For Jonckheere Terpstra patterned rank test, Gnu R open source software using the SAGx package was used (http://www.r-project.org). Used tests are indicated at the table and figure legends. According to the guidance for publication in the *BJP* (Curtis et al., 2015), after ANOVA, post hoc tests were only performed if *F* had achieved the significance level of *P* < .05. For animal experimentation, sample size was calculated for the primary endpoint (clinical disease severity 1, 2, 3, and 4 weeks after randomization) using SigmaPlot with the following assumptions: minimum detectable difference of 50%, *SD* 25%, power 90%, α of 5%, and ANOVA with eight groups (two interventions, four time points). This requires a sample size of 11 per group.

### 2.11 Materials

The antibodies used were as follows. Rozanolixizumab (UCB7665) is an anti‐human FcRn IgG4P mAb under investigation in clinical trials (Kiessling et al., 2017). A pharmacologically equivalent murinised anti‐mouse FcRn IgG1 antibody (4470) was used in a preclinical mouse model of an autoantibody‐driven autoimmune disease. Generation of the anti‐mouse FcRn antibody 4470 and an isotype matched control antibody has recently been described (Smith et al., 2019).

### 2.12 Nomenclature of targets and ligands

Key protein targets and ligands in this article are hyperlinked to corresponding entries in http://www.guidetopharmacology.org, the common portal for data from the IUPHAR/BPS Guide to PHARMACOLOGY (Harding et al., 2018), and are permanently archived in the Concise Guide to PHARMACOLOGY 2019/20 (Alexander, Fabbro et al., 2019; Alexander, Kelly et al., 2019).

## 3 RESULTS

### 3.1 Efficacy of the anti‐FcRn antibody

To validate the efficacy of the 4470 anti‐FcRn antibody in an additional mouse strain and to investigate if the antibody also reduces serum IgG concentrations in mice immunized with TiterMax®, IgG serum concentrations were determined in B6.s mice treated with (a) isotype antibody alone, (b) 4470, (c) TiterMax® and isotype antibody, and (d) TiterMax® and 4470, at the start of treatment and 8 weeks thereafter. All data were normalized to the IgG serum concentrations at the start of treatment. In all groups treated with isotype antibody, a significant increase from pretreatment IgG serum concentration was noted, while this was not observed for mice treated with anti‐FcRn. In addition, in untreated, as well as in TiterMax®‐injected mice, anti‐FcRn treatment led to a significant decrease of IgG at Week 8. Hence, as expected, the 4470 anti‐FcRn antibody reduced total IgG concentrations in B6.s mice, in both untreated and TiterMax® injected mice.

### 3.2 Treatment of mice with clinically manifest EBA with anti‐FcRn improves disease manifestation

**Figure 1:**
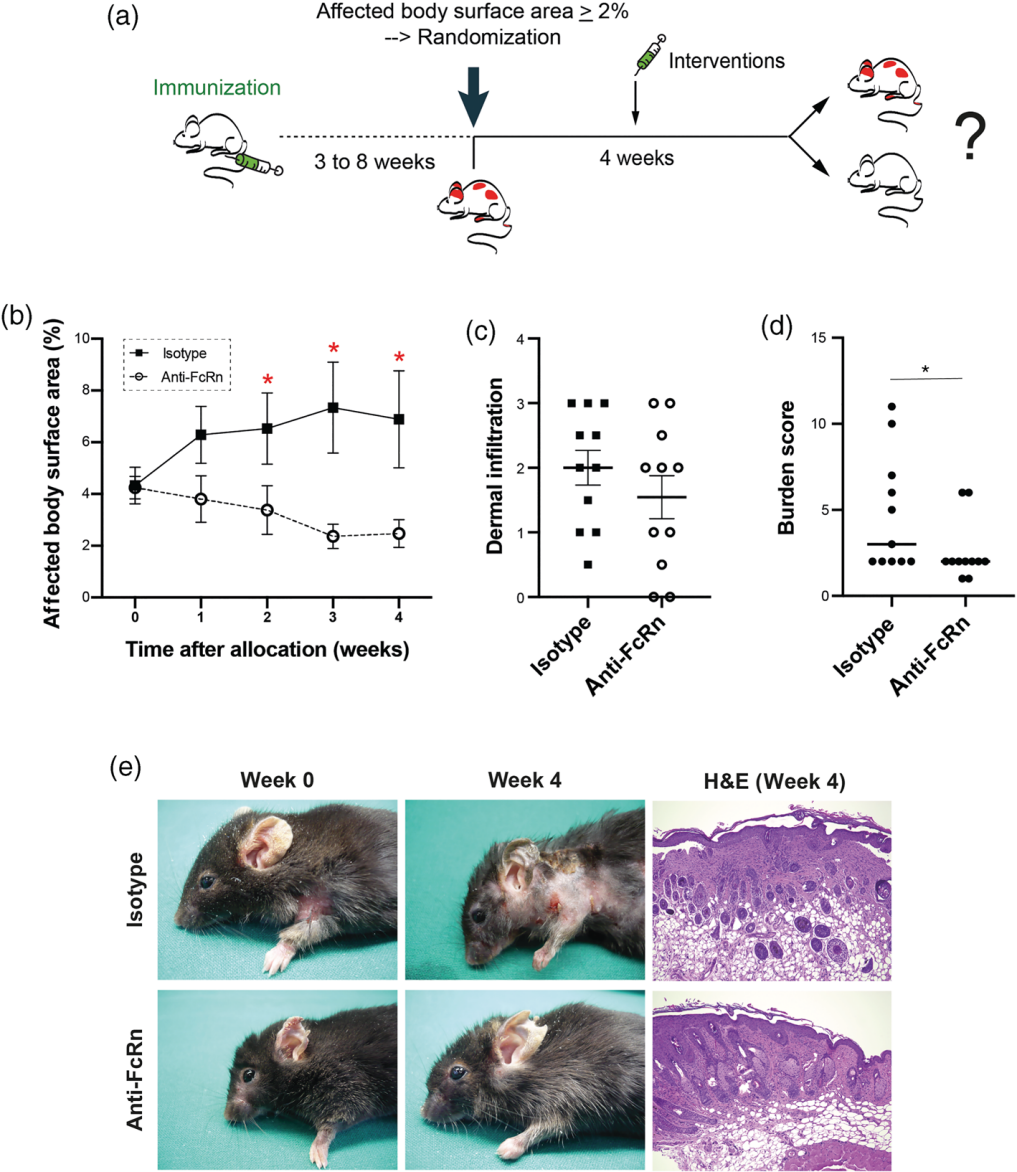
Anti‐FcRn treatment improves already clinically manifest experimental EBA. (a) B6.s mice were immunized with mCOL7^vWFA2^ for induction of experimental EBA. Three to 8 weeks after immunization, mice were weekly clinically evaluated. If, in an individual mouse, 2% or more of the body surface area was affected by EBA lesions, it was randomized to either isotype or anti‐FcRn antibody treatment. Treatments were carried out for 4 weeks, and clinical disease severity, expressed as affected body surface area, was evaluated weekly. (b) At randomization (Week 0), clinical disease severity, expressed as percentage of affected body surface area, was identical in isotype‐ and anti‐FcRn antibody‐treated mice. In isotype‐treated mice, clinical EBA symptoms worsened during the 4‐week treatment period, while it improved in anti‐FcRn treated mice. Starting from Week 2, compared to mice injected with isotype antibody, a significant lower affected body surface area was observed in anti‐FcRn‐treated mice until the end of the experiment. Data shown are the means ± SEM, from 11 mice per group. *P< .05, significantly different as indicated; two‐way ANOVA with Bonferroni *t* test for pairwise multiple comparisons. (c) Dermal infiltration was semi‐quantitatively evaluated in biopsies from lesional skin at the end of the experiment. No difference in infiltration among the two groups was noted; Student's *t* test. Data shown are the individual values with means ± SEM, from 11 mice per group. (d) Burden score (excluding EBA skin lesions) of the mice treated with isotype or anti‐FcRn antibody at Week 4 after randomization. The graph shows each score (dots) and the median (line). Data shown are the individual values with medians, from 11 mice per group. *P< .05, significantly different as indicated; Mann–Whitney test. (e) Representative clinical images of the same mice at randomization (Week 0) and at the end of the treatment (Week 4) receiving either isotype or anti‐FcRn antibody. Representative H&E stained skin biopsies from lesional skin, obtained at the end of the treatment period

To evaluate the effects of anti‐FcRn treatment in already manifest AIBD, we induced experimental EBA in mice by immunization. In order to duplicate the experimental set‐up of clinical trials, only mice that met pre‐defined disease severity were treated with anti‐FcRn or the isotype antibody. To prevent observer bias, the investigator scoring the mice for the primary endpoint (clinical disease) was unaware of the applied treatment (Figure 1a). At randomization, mice of both groups presented with similar disease severity (Figure 1b). Furthermore, time taken after immunization to reach the required disease severity was also identical (4.6 ± 1.0 weeks for isotype‐, and 4.9 ± 1.0 weeks for anti‐FcRn‐treated mice). In isotype‐treated mice, clinical disease manifestation, expressed as body surface area affected by EBA skin lesions, increased during the 4‐week experimental perioid. In this group, the maximum extent of affected body surface area, 7.3% ± 1.8% (representing a 1.7‐fold increase in involved body area), was achieved by Week 3. In contrast, mice receiving anti‐FcRn antibody were reported to be less severely affected at Weeks 2, 3, and 4 (Figure 1b,d), and complete remission was noted in some individual animals (Table 1). At the end of the experiment, lesional skin biopsies were obtained from both isotype‐ and anti‐FcRn‐treated mice, to validate, by histology, the presence of EBA. As expected, subepidermal blistering and dermal infiltrations were present in both groups (Figure 1c,e).

**Table 1:** Anti‐FcRn treatment induces remission in individual mice

	Progression	Partial Remission	Complete Remission
Isotype	7	4	0
Anti‐FcRn	2	6	3

*Note.* Mice with clinical EBA manifestations were randomized to either isotype antibody or anti‐FcRn treatment. Treatments were carried out over 4 weeks. Treatment outcomes were defined as progression (body surface area affected by EBA increased from Week 1 to Week 4), partial remission (body surface area affected by EBA decreased from Week 1 to Week 4, and total affected body surface area is ≥1%), or complete remission (body surface area affected by EBA decreased from Week 1 to Week 4, and total affected body surface area is <1%). Affected body surface area for mice in “complete remission” were 0.03% and 0.6% in two mice. The difference in proportions (progression versus remission [partial and complete]) is significant (*P* < .05, chi‐squared test).

There are some reports in the mouse linking genetic deficiency of either the FcRn α chain or β2 microglobulin to the induction of autoantibodies and, indeed, to the spontaneous development of arthritis. To address this possible issue, we recorded the well‐being of the animals during the experiment and recorded any adverse events during the treatment with either isotype or anti‐FcRn antibody. The overall burden of the mice in the treatment group was mild in most cases (scores <10, Table S1). The burden of the mice in the anti‐FcRn‐treated group was lower compared to the isotype antibody treated group (Figure 1d). In addition, ANA were determined in both groups at the end of the treatment period. In contrast to sera from lupus‐prone NZM2410/J mice from a previous study who had developed proteinuria (Vorobyev et al., 2019), which were all ANA positive, no ANA were detected in either isotype‐ or anti‐FcRn‐treated mice.

### 3.3 Pharmacological FcRn inhibition reduces circulating autoantibody concentrations as well as tissue bound IgG deposits in the skin

**Figure 2:**
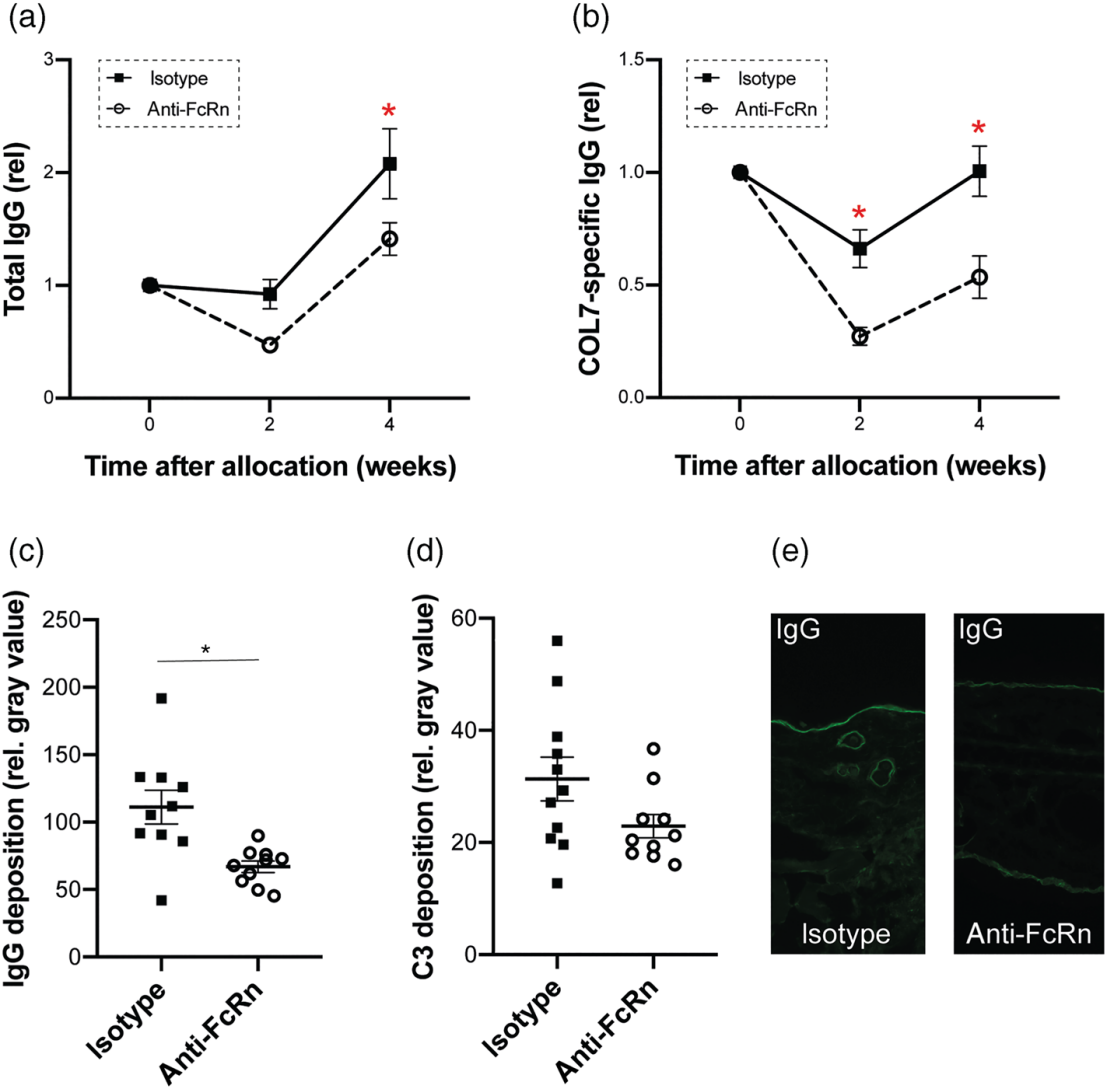
Anti‐FcRn treatment reduces circulating anti‐COL7 IgG concentrations and decreases IgG deposition in the skin of mice with experimental EBA. Isotype or anti‐FcRn antibody treatment was initiated if, in an individual mouse, 2% or more of the body surface area was affected by EBA lesions. At the start of treatment, as well as 2 and 4 weeks thereafter, serum was obtained for determination of total and COL7‐specific IgG. (a) In both groups, total IgG concentrations increased during the 4‐week treatment period. This increase was less pronounced in mice treated with anti‐FcRn. Data shown are the means ± SEM, from 9‐10 mice per group, after removal of outliers using ROUT, accounting for the unequal *n*. *P< .05, significantly different from anti‐FcRn; two‐way ANOVA with Bonferroni *t* test for pairwise multiple comparisons.. (b) Concentrations of COL7‐specfic IgG remained relatively constant in isotype antibody treated mice, while anti‐FcRn antibody treatment reduced the specific autoantibody concentration by approximately 50%. Data shown are the means ± SEM, from 9‐10 mice per group, after removal of outliers using ROUT, accounting for the unequal *n*. *P< .05, significantly different from anti‐FcRn; two‐way ANOVA with Bonferroni *t* test for pairwise multiple comparisons. In (c) the deposition of IgG, but not of C3 (d), at the dermal–epidermal junction junction was reduced in mice treated with anti‐FcRn antibody, compared with mice injected with isotype control antibody. Data shown are the individual values with means ± SEM, from 9‐12 mice per group, after removal of outliers using ROUT. *P< .05, significantly different as indicated; *t* test (IgG) or rank sum test (C3). (e) Representative images of direct IF microscopy for IgG from peri‐lesional skin biopsies obtained at the end of the experiment

At this point, therapeutic efficacy of anti‐FcRn treatment in experimental EBA had been established. Based on previous observations, this effect could either be modulated by a decrease of IgG half‐life (Stapleton et al., 2015), by inhibiting the binding of myeloid cells to the immune complexes (Vidarsson et al., 2006), or by a combination of both effects. To address the effects of FcRn inhibition on total and COL7‐specific IgG concentrations, their respective serum concentration was determined at randomization (Week 0), as well as 2 and 4 weeks thereafter. Immunization of mice with COL7^vWFA2^ not only led to the induction of a COL7^vWFA2^‐specific IgG response, but in parallel also induced an approximate twofold increase in total IgG in isotype‐antibody treated mice. In animals injected with 4470, both total and COL7^vWFA2^‐specific IgG serum concentrations were significantly lower compared to the isotype antibody‐treated mice (Figure 2a,b). Lower concentrations of circulating autoantibodies targeting COL7^vWFA2^ were accompanied by reduced deposition of these autoantibodies in the skin, as shown by a reduced linear IgG deposition along the dermal–epidermal junction by direct IF microscopy in mice treated with the anti‐FcRn antibody (Figure 2c,e). Hence, compared to isotype antibody‐treated mice, the density of tissue‐bound immune complexes in the skin was lower in mice following inhibition of FcRn.

### 3.4 Anti‐FcRn treatment does not alter neutrophil CD62L expression in vivo

**Figure 3:**
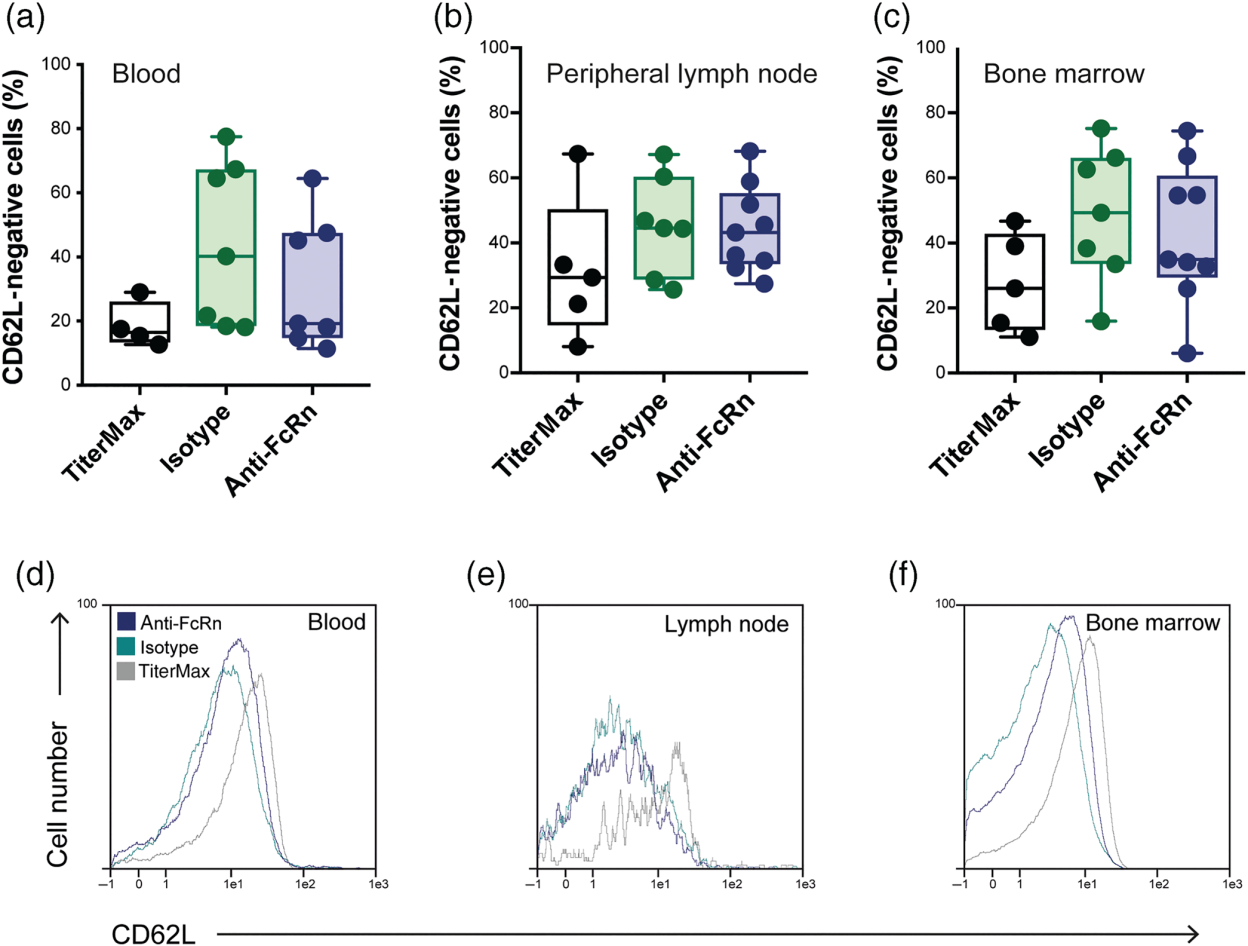
Anti‐FcRn treatment does not alter neutrophil CD62L function in vivo. After 4 weeks of treatment with either isotype or anti‐FcRn antibody, indicated organs from mice with experimental EBA were obtained for isolation of neutrophils, followed by flow cytometry. Mice injected with TiterMax® alone served as negative controls. (a–c) Percentage of CD62L^−^ CD45^+^, CD1b^+^ Gr‐1^+^, and Ly6G^+^ singlet cells was determined. No differences in neutrophil activation was detected in the three investigated organs. Data are shown as individual values in box and whisker plots with medians, quartiles and ranges, from seven to eight mice per group; unequal sample sizes are due to technical issues (i.e., clotting). For normally distributed data, *t* test was used for statistical analysis, and for non‐equally distributed data, rank sum test was applied. (d–f) Representative FACS images are shown in panels a–c

In addition to its expression on epithelial cells, placental syncytiotrophoblasts, and endothelial cells, where the FcRn functions to transport IgG across mucosal cells, from mother to fetus, and regulates IgG half‐life, respectively (Stapleton et al., 2015), FcRn is also expressed by monocytes, NK cells, and neutrophils. Upon neutrophil activation, FcRn locates at the plasma membrane of neutrophils. Functionally, neutrophil‐expressed FcRn promotes phagocytosis which is triggered by binding of IgG‐opsonized bacteria to the neutrophils (Vidarsson et al., 2006). As immune complex activation of neutrophils promotes tissue injury in several autoimmune diseases, including experimental EBA (Koga et al., 2019; Ludwig et al., 2017), we next investigated if anti‐FcRn changes neutrophil CD62L expression in EBA‐affected mice. For this purpose, expression of CD62L, which corresponds to the activation status (Rosales, 2018), on neutrophils from different compartments (blood, peripheral lymph node, and bone marrow) was assessed at the end of the 4‐week treatment period. No significant difference in CD62L expression on neutrophils was noted between isotype‐ or anti‐FcRn‐treated mice in any of the investigated compartments using ANOVA. However, the Jonckheere Terpstra patterned rank test, which allows detection of trends among different groups, showed a significant trend towards lower CD62L^−^ neutrophils in the blood in TiterMax® immunized mice, indicating that induction of experimental EBA leads to an activation of neutrophils within the circulation (Figure 3).

### 3.5 Immune complex‐induced activation of neutrophils is not influenced by anti‐FcRn treatment

**Figure 4:**
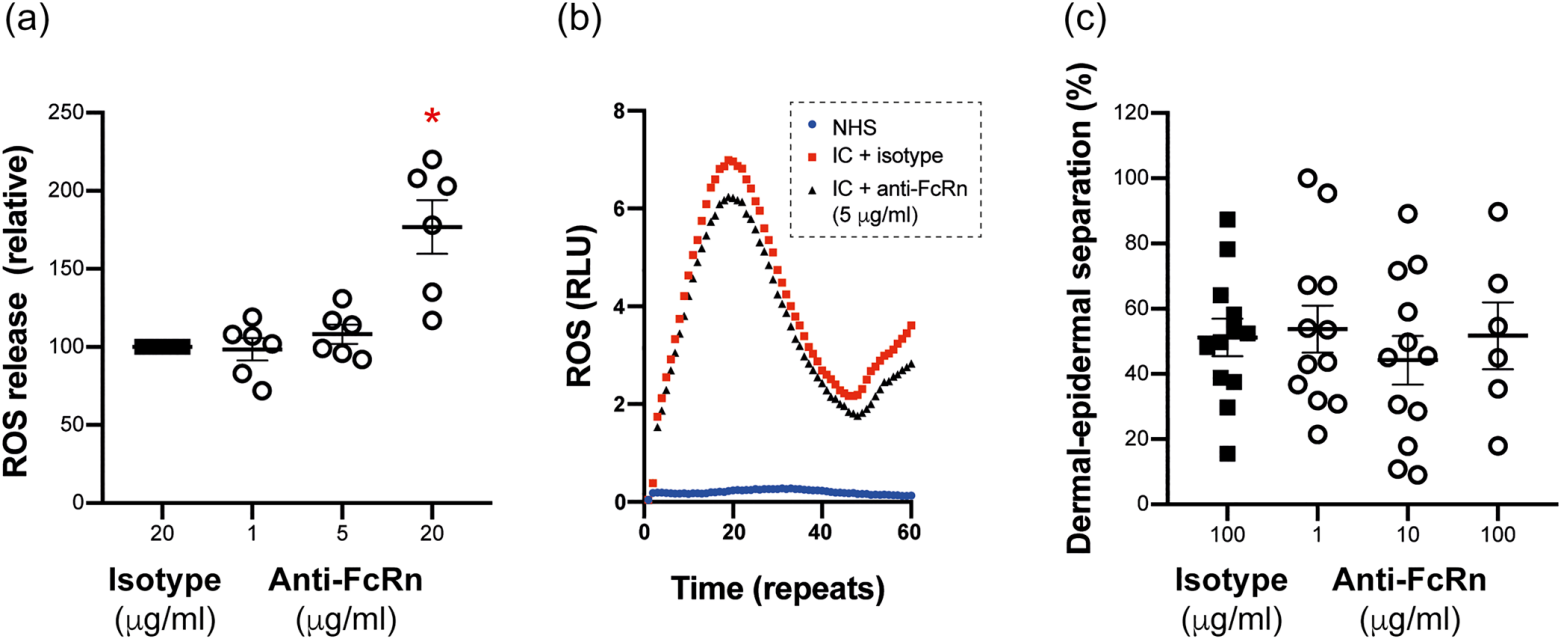
Blockade of the FcRn did not affect immune complex‐induced neutrophil activation. (a) Neutrophils were stimulated with immune complexes, and their activation was determined by the cumulative release of ROS over time. Data shown are the individual values with means ± SEM, from 6 samples per group. *P< .05, significantly different from isotype; ANOVA with Bonferroni *t* test for multiple comparisons. (b) Representative results of one ROS‐release experiment. Time is measured in repeats, whereby one repeat corresponds to approximately 1 s. (c) Dermal–epidermal separation was induced on skin cryosections by incubating these with anti‐COL17 IgG and neutrophils. Blockade of the FcRn had no effect on the magnitude of dermal–epidermal separation. If sections were incubated with normal IgG, instead of anti‐COL17 IgG, and neutrophils, dermal–epidermal separation ranged between 0% and 5%. Data shown are the individual values with means ± SEM, from 12 samples per group, with the exception of the 100 μg·ml^−1^ dose, where *n* = 6. ANOVA, with Bonferroni *t* test for multiple comparisons, was used to test for statistical difference from isotype

We next evaluated the effects of the FcRn on neutrophil activation in vitro. For this, neutrophils were activated with immune complexes in the absence or presence of the anti‐FcRn antibody 4470. Compared to neutrophils incubated with isotype‐antibody, lower doses of 4470 had no effect on neutrophil activation (expressed as release of ROS over time), while at higher concentrations, anti‐FcRn led to an enhanced ROS release from the neutrophils (Figure 4a,b). This suggests that neutrophil activation leads to enhanced surface expression of the FcRn (Vidarsson et al., 2006). In turn, anti‐FcRn can bind to the cell surface and may lead to immune complex enhanced neutrophil activation. However, rapid internalization of this complex limits the duration of effect. This assumption is supported by the unaltered dermal–epidermal separation on skin cryosections incubated with anti‐COL7 IgG and neutrophils and anti‐FcRn at higher doses (Figure 4c).

## 4 DISCUSSION

Taking our results together, we have here demonstrated the efficacy of anti‐FcRn treatment in experimental murine EBA and concluded that the therapeutic effects of anti‐FcRn treatment are associated with a reduced concentration of circulating and tissue‐bound autoantibodies targeting COL7.

Previous data had indicated that FcRn is involved in the pathogenesis of autoantibody‐mediated diseases: First, genetic alterations of the Fc‐molecule that alter its binding to FcRn, have been shown to be associated with autoantibody‐mediated disease—specifically in pemphigus, where the p.Arg435his variation of IgG3 that has a high affinity to the FcRn is associated with disease susceptibility (Recke et al., 2018). Second, in antibody transfer models of pemphigus, bullous pemphigoid, EBA, or myasthenia gravis, FcRn‐ or β2 microglobulin‐deficient mice were completely or partly protected from induction of experimental disease (Li et al., 2005; Liu et al., 1997; Liu et al., 2007; Sesarman et al., 2008). Third, in the K/BxN serum transfer model of arthritis, prophylactic blockade of the FcRn by Fc‐engineered antibodies to block FcRn (Abdegs) impaired arthritis development, and in short‐term, quasi‐therapeutic settings also led to a delayed disease progression (Patel et al., 2011). Similar results were observed in an antibody transfer‐enhanced model of murine experimental autoimmune encephalomyelitis (Patel et al., 2011) and in an antibody transfer‐induced model of immune thrombocytopenia (Smith et al., 2019).

However, genetic deficiency of either the FcRn α chain or β2 microglobulin has also been reported to enhance the production of certain autoantibodies in the mouse (Singh, Yang, Kim, & Halder, 2013) and to lead to spontaneous arthritis development (Kingsbury et al., 2000). Furthermore, application of FcRn‐targeted treatments in long‐term therapeutic settings had not been performed so far, although no adverse events were detected in a non‐human primate study where animals were administered for long periods of time with high doses of an anti‐FcRn monoclonal antibody (Smith et al., 2018). Hence, the long‐term effects of pharmacological FcRn inhibition in therapeutic settings of autoimmune diseases are currently not known. To investigate if long‐term, pharmacological blockade of the FcRn could have therapeutic effects in a prototypical autoantibody‐mediated disease, we induced experimental EBA by immunization. Following development of a disease of defined severity, mice were randomized to a 4‐week treatment period, comparing the effects of anti‐FcRn or isotype antibody on further disease development.

We here clearly document that anti‐FcRn antibody treatment is therapeutically active in experimental EBA. Overall, clinical disease manifestation improved in over 80% of the mice, and the affected body surface area decreased by almost 50%. Disease remission was observed in nine of 11 mice, and in three, only minimal skin lesions were present at the end of the 4‐week treatment period. In contrast, clinical disease progressed in mice injected with an isotype control antibody, although spontaneous partial, but never complete, remission was observed in three of 11 mice. Hence, targeting FcRn is indeed a promising therapeutic approach for the treatment of autoantibody‐mediated diseases including EBA. This approach is currently being evaluated in phase II clinical trials in patients with pemphigus (NCT03334058 and NCT03075904) and other autoantibody‐mediated disorders (NCT03075878, NCT03052751, and NCT02718716). The rationale for FcRn inhibition in diseases with a clearly different pathogenesis (i.e., pemphigoid versus pemphigus) is based on the finding that the (different) autoantibodies reach their target antigen through the bloodstream, where the half‐life is controlled by the FcRn.

FcRn‐targeting antibodies alone may be insufficient for many of these diseases, because, with a few exceptions, immunoadsorption/plasmapheresis is insufficient to induce long‐term remission in most autoantibody‐mediated diseases (Meyersburg, Schmidt, Kasperkiewicz, & Zillikens, 2012). Immunoadsorption in bullous pemphigoid patients induces rapid and long‐lasting remission, even in patients receiving no other medications (Hübner et al., 2018). Hence, in bullous pemphigoid, anti‐FcRn antibody may be a preferred treatment option. For most other AIBD, we believe that anti‐FcRn treatment, like immunoadsorption, will induce rapid improvement, but additional immunosuppressive treatment would be needed to induce long‐term remission. One such candidate may be anti‐CD20 therapy (Joly et al., 2017), but due to the IgG clearing effect of anti‐FcRn treatment, anti‐CD20 mAb would also be cleared rapidly if administered concurrently. As an alternative, B‐cell inhibitors such as https://www.guidetopharmacology.org/GRAC/ObjectDisplayForward?objectId=1948) inhibitors may be used. BTK inhibitors are currently in phase II clinical trials for pemphigus (NCT02704429).

Based on these insights into FcRn biology and FcRn‐targeting therapies in a prototypical autoantibody‐mediated disease, clinical trials evaluating FcRn‐targeting therapies are merited in pemphigoid diseases and may enable an additional treatment option for the still unmet (Lamberts et al., 2019) medical need. As pemphigoid diseases are prototypical autoantibody‐mediated diseases, it is tempting to speculate that targeting the FcRn may also be of benefit in AIBD and other autoantibody‐mediated diseases.

## AUTHOR CONTRIBUTIONS

A.K., M.H., K.B., B.S., and P.W. performed experiments, and B.S., P.W., A.S., R.J.L., and E.S. conceived the study. All authors interpreted the data and wrote and revised the manuscript.

## CONFLICT OF INTEREST

In the last 3 years, R.J.L. has received research funding from Almirall, True North Therapeutics, UCB Pharma, ArgenX, TxCell, Topadur, Incyte, and Admirx and fees for consulting or speaking from ArgenX, Immunogenetics, Novartis, and Lilly. E.S. has received research funding from Almirall, UCB Pharma, ArgenX, TxCell, Incyte, Novartis, Euroimmun, and Admirx and fees for consulting or speaking from ArgenX, UCB, TxCell, Novartis, Fresenius, and Almirall. A.S. is an employee of UCB, and P.W. and B.S. are former employees of UCB. All other authors declare no conflict of interest.

## DECLARATION OF TRANSPARENCY AND SCIENTIFIC RIGOUR

This Declaration acknowledges that this paper adheres to the principles for transparent reporting and scientific rigour of preclinical research as stated in the *BJP* guidelines for https://bpspubs.onlinelibrary.wiley.com/doi/full/10.1111/bph.14207, https://bpspubs.onlinelibrary.wiley.com/doi/full/10.1111/bph.14208, and https://bpspubs.onlinelibrary.wiley.com/doi/full/10.1111/bph.14206, and as recommended by funding agencies, publishers and other organisations engaged with supporting research.

## Supporting information

Table S1 The sum of all adverse effects was added to calculate the burden of disease. A score of <10 corresponds to mild burden, scores between 10 and < 20 are considered moderate, while scores ≥20 are considered severe. Mice are killed if the cumulative score is 20 or more, of if the body surface area affected by inflammatory skin lesions is 20% or more, at any time during the experiment.
