# Supplementary material for: Treatment with anti‐neonatal Fc receptor (FcRn) antibody ameliorates experimental epidermolysis bullosa acquisita in mice
Source: Br J Pharmacol. 2020 Mar 6;177(10):2381–92. doi: 10.1111/bph.14986 (PMC7174883; doi:10.1111/bph.14986)
Supplement: Supplementary file 1 — Table S1 The sum of all adverse effects was added to calculate the burden of disease. A score of <10 corresponds to mild burden, scores between 10 and < 20 are considered moderate, while scores ≥20 are considered severe. Mice are killed if the cumulative score is 20 or more, of if the body surface area affected by inflammatory skin lesions is 20% or more, at any time during the experiment. [file BPH-177-2381-s001.docx]

| **I Body weight** | **value** |
| --- | --- |
| unaffected or rise | 0 |
| Weight reduction <5 % compared to day 0 of the experiment | 1 |
| Weight reduction 5-10 % compared to day 0 of the experiment | 5 |
| Weight reduction 11-20 % compared to day 0 of the experiment | 10 |
| Weight reduction >20 % compared to day 0 of the experiment | 20 |
| **II General condition** |  |
| Coat smooth, shiny, body openings clean, eyes clear, shiny | 0 |
| Fur blunt, disorderly, unkempt body openings, eyes cloudy, increased muscle tone | 1 |
| Dirty coat, sticky or wet body openings, abnormal posture, eyes cloudy, increased muscle tone | 5 |
| Dirty coat, sticky or damp body openings, abnormal posture, eyes cloudy; high muscle tone | 10 |
| Cramps, paralysis (trunk muscles, extremities),wheezing, animal feels cold | 20 |
| **III Spontaneous behavior** |  |
| Normal behavior (sleeping, reaction to blowing and touching, curiosity, social contacts) | 0 |
| Small deviations from the normal behavior | 1 |
| Unusual behavior, impaired motor function or hyperkinetics (noticeable scratching over 2 of 10 min) | 5 |
| Self-isolation, lethargy, pronounced hyperkinetics or behavioral stereotypies, coordination disorders hyperkinetics (noticeable scratching behavior over 5 of 10 min) | 10 |
| Repeated pain sounds when grasping, self-amputation (autoaggression, autotomy) | 20 |
| **IV Clinical findings (measurements of temperature and respiration only if abnormalities are observed under II and III)** |  |
| Temperature, respiration and pulse normal, extremities warm, mucous membranes well supplied with blood | 0 |
| Small deviations from the normal situation | 1 |
| Temperature deviation 1 - 2 °C, respiration and pulse + 30% | 5 |
| Temperature deviation> 2 °C, respiration / pulse +/- 50% | 10 |
| Temperature, respiration and pulse normal, extremities warm, mucous membranes well supplied with blood | 20 |
| **Sum** | **0-80** |

**Supplemental table 1.** Adverse events score.

The sum of all adverse effects was added to calculate the burden of disease. A score of <10 corresponds to mild burden, scores between 10 and <20 are considered moderate, while scores >20 are considered severe. Mice are euthanized if the cumulative score is 20 or more, of if the body surface area affected by inflammatory skin lesions is 20% or more, at any time during the experiment.
